# Location-Based Augmented Reality for Cultural Heritage Communication and Education: The Doltso District Application

**DOI:** 10.3390/s23104963

**Published:** 2023-05-22

**Authors:** Alexandros Kleftodimos, Athanasios Evagelou, Amalia Triantafyllidou, Magdalini Grigoriou, Georgios Lappas

**Affiliations:** 1 Department of Communication and Digital Media, University of Western Macedonia, 52100 Kastoria, Greece; atriantafylidou@uowm.gr (A.T.); glappas@uowm.gr (G.L.); 2 The Center for Education for the Environment and Sustainability of Kastoria, 52100 Kastoria, Greece; evagel@sch.gr (A.E.); magdalene@sch.gr (M.G.)

**Keywords:** location-based applications, augmented reality, game-based learning, cultural heritage communication, cultural heritage education

## Abstract

Location-based Augmented Reality applications are increasingly used in many research and commercial fields. Some of the fields that these applications are used are recreational digital games, tourism, education, and marketing. This study aims to present a location-based augmented reality (AR) application for cultural heritage communication and education. The application was created to inform the public, especially K12 students, about a district of their city with cultural heritage value. Furthermore, Google Earth was utilized to create an interactive virtual tour for consolidating the knowledge acquired by the location-based AR application. A scheme for evaluating the AR application was also constructed using factors suitable for location-based applications: challenge, educational usefulness (knowledge), collaboration, and intention to reuse. A sample of 309 students evaluated the application. Descriptive statistical analysis showed that the application scored well in all factors, especially in challenge and knowledge (mean values 4.21 and 4.12). Furthermore, structural equation modeling (SEM) analysis led to a model construction that represents how the factors are causally related. Based on the findings, the perceived challenge significantly influenced the perceived educational usefulness (knowledge) (b = 0.459, sig = 0.000) and interaction levels (b = 0.645, sig = 0.000). Interaction amongst users also had a significant positive impact on users’ perceived educational usefulness (b = 0.374, sig = 0.000), which in turn influenced users’ intention to reuse the application (b = 0.624, sig = 0.000).

## 1. Introduction

Location-based augmented (LBAR) applications have existed for quite some time, but their use for various communication purposes has become widespread after the advent of two popular augmented-reality location-based games, namely Pokemon Go and Ingress, created by Niantic in 2014 and 2016, respectively. Location-based augmented reality apps (also called location-aware augmented reality) are currently used in a number of fields, such as entertainment, education, and marketing. An application can also be used to fulfill more than one purpose. For example, an LBAR mobile application can be used for tourism marketing, but at the same time, it can also provide information that educates visitors about a tourist destination.

Location-based augmented reality applications typically run on mobile devices (smartphones or tablets) and make use of the GPS sensor of the mobile device to guide users to specific locations. When these locations are reached, digital content is activated. Digital content can be in many forms, such as text, video, 2D and 3D graphics, and animations. Location-based augmented reality can also be combined with image-based augmented reality to superimpose the real world with digital content when specific locations are reached, and the camera of the mobile device is pointed at objects from the real environment (e.g., constructions, monuments, etc.) [1].

Cultural heritage communication is increasingly taking advantage of innovations in digital technologies, and LBAR applications can be utilized for creating informative cultural tours. Using LBAR applications, users are prompted to visit cultural heritage monuments and places where significant historical events have occurred. When the users get close to these locations, digital content appears on the screens of their mobile devices, informing them about various aspects of these locations, such as historical facts, architectural and artistic trends related to human constructions, etc. Applications designed for augmenting the environments with cultural heritage content are numerous. Overlaying the physical world with historical information is present in many initiatives (e.g., [2,3,4]).

This study aims to present a location-based augmented reality application for cultural heritage communication and education and the considerations that have taken place in the pre-production phase. The study also presents a Google Earth interactive application intended for use after the augmented reality experience to refresh and strengthen the knowledge acquired during the cultural tour. Another aim of this study is to construct a suitable evaluation scheme for assessing the efficiency of the location-based AR application and to evaluate multivariate causal relationships among various factors using structural equation modeling. The factors which were used in the evaluation were outdoor activity, challenge, ease of use, interaction/collaboration, knowledge, and intention to reuse.

To summarize, the research objectives of this study were the following:

- To create and present a location-based AR experience for cultural heritage education that is engaging to learners and could act as an example for educators wishing to employ AR in their cultural education tours.
- To present the steps for developing such applications, giving emphasis to the decisions taken in the pre-production phase.
- To create and present a VR Google Earth project that would be used after the AR experience to consolidate the acquired knowledge.
- To construct a suitable evaluation scheme based on past research for assessing the factors related to a location-based AR experience.
- To evaluate multivariate causal relationships among various factors using structural equation modeling (SEM).

This article is organized in the following way: A brief literature review is provided in Section 2. The district of Doltso and the related educational program are described in Section 3, and materials and methods are presented in Section 4. Continuing the augmented reality application is presented in Section 5, together with the steps followed to develop the application. Particular emphasis is given in this section to the considerations taken place in the pre-production phase.

Then a Google Earth interactive application that was created to refresh the knowledge obtained from the AR tour is presented in Section 6. The process for evaluating the AR application is described in Section 7, and the evaluation results are presented in Section 8, together with a discussion on the findings, limitations, and recommendations for future research. The paper concludes in Section 9.

## 2. Related Work

Augmented Reality (AR) has a long history. The following subsection presents some important milestones in the AR evolution.

### 2.1. Important Milestones and Types of AR

The first AR technology, an AR head-mounted display, was developed in 1968 at Harvard [5] by a scientist called Ivan Sutherland. The head-mounted display was called “The Sword of Damocles”. AR technology evolved through the efforts and scientific achievements of academia and the computer industry in the decades to come. In 1974, a laboratory called Videoplace was built. Videoplace combined projectors with video cameras to create an artificial reality. However, it was not until the 1990s that AR experienced significant growth. In 1990, the term augmented reality was coined by a Boeing researcher named Tom Caudell, and in 1992, the first immersive and fully functional augmented reality system called “Virtual Fixtures” was developed by Luis Rosenberg. A few more important advancements took place in the 1990s. In 1994, the first augmented reality Theater production was created, titled “Dancing in Cyberspace”, featuring real acrobats dancing within and around virtual objects on stage. In 1998, the 1st & Ten line system was introduced by SportsVision. The system augmented the live broadcast display of American football fields with yellow lines to help the viewers understand the game’s progression. In 1999, NASA created a hybrid synthetic vision system for their X-38 spacecraft, an experimental vehicle, to assist navigation [6].

In the following decades, the growth of AR was even more remarkable. A significant milestone in AR evolution was in 2000 when an open-source library called AR toolkit was released, providing developers with tools for rapid augmented reality application development. In 2009, Esquire magazine introduced AR in print media, and in 2013, Volkswagen created the MARTA app, which gave technicians step-by-step instructions by augmenting the vehicle parts with text and animations. In 2014, Google introduced its augmented reality glasses, and in 2016, Microsoft started shipping its AR head-mount device HoloLens [6].

Quite a few categorizations have been presented throughout the evolution of AR. One categorization is based on the devices that enable an AR experience. AR can be achieved through wearable and non-wearable devices. Wearable devices include headsets and helmets, and non-wearable devices include mobile devices (smartphones, tablets, etc.), stationary devices (TVs, PCs, etc.), and head-up displays (integrated or retrofitted) [7]. Another taxonomy concerns the way the in which the AR experience is triggered. Marker-based AR, also referred to as image-based AR, relies on visual markers (e.g., QR codes, printed images, real-world objects, etc.) to trigger the experience. Markerless AR, on the other hand, does not rely on physical markers. Instead, mobile device cameras and other sensors are used to detect and track the user’s environment and determine the place where the virtual content will appear. Two common types of Markerless AR are projection-based AR and location-based AR. Projection-based AR uses projectors to display multimedia content (e.g., 3D images) onto flat two-dimensional surfaces, such as building walls. In location-based AR, the content is fixed to a specific physical location. Mobile devices with GPS sensors are the most common way to identify specific locations and superimpose the real environment with multimedia content through the mobile device camera. The multimedia content can be in the form of text, images (3D and 2D), sound, videos, and animations.

### 2.2. AR in Education

Today AR is being utilized in many industry sectors. For example, tourism is an industry sector exploiting AR technologies’ potential for marketing and educational purposes. Education is a sector that also benefits from AR, and by exploring the literature, one can find a wide variety of applications that have been applied at all levels of education.

For example, AR applications are being developed for primary education [8,9,10], secondary [11,12], and tertiary education [13,14]. Augmented reality is also used for adult learner education and special needs education [15,16]. AR has also been used to support a wide range of subjects, as can be seen in a recent literature review [17]. These subjects include language learning [18], mathematics [8,19,20], chemistry [21,22], computer science [23,24], engineering [25], history [2,26], physics [27], STEM subjects [14,28], biology [29,30], and environmental education [31].

A relatively recent systematic review and meta-analysis of AR in educational settings shows a remarkable increase in research studies between 2012 and 2018 [32]. According to this review, many studies reported the advantages of using AR in educational settings. These advantages refer to positive effects on learning gains and student attitudes, such as improvement of academic performance, motivation, creativity, and autonomy. However, some disadvantages were also reported in the studies examined in this review paper. The most reported disadvantage was the complexity of using AR, especially when the target group was children. New technology, such as AR, can be complex, especially when the users do not have technological expertise. The rest of the issues encountered are also associated with this complexity. Technical difficulties were reported by teachers when using AR in their classrooms. Another reported problem was multitasking. Students expressed that these applications demand too much attention, which can cause students to omit essential instructions. Finally, resistance from teachers has also been reported as an issue. Some teachers prefer having total control over the content, despite the benefits of AR applications in learning gains and motivation.

AR has also proved successful in cultural heritage education and communication [33,34,35]. AR is a valuable technology that can enhance a visitor’s experience and knowledge about cultural heritage, and there are several examples of initiatives in this field (e.g., [36,37,38]). Advancements in AR technology, moving from marker-based to marker-less and GPS-triggered overlays, have made AR even more compelling for this field. In addition, Jung et al. [39] conducted a study on cultural differences in AR in heritage sites and found that Western visitors strongly desire to escape reality through AR applications.

A quick analysis of the scientific literature reveals that most AR projects are based on a marker-based approach. For example, a systematic mapping review of STEM augmented reality applications in higher education [14] revealed the scarcity of marker-less AR and location-based applications in the specific field.

Despite its limited use, location-based AR has great potential in cultural heritage communication and education. Location-based AR can help users learn historical facts and other aspects of cultural heritage through visits to urban environments, archaeological sites, and museums. In the following sub-section, some examples of research efforts that focus on this specific topic are briefly presented.

### 2.3. Location-Based AR Initiatives for Cultural Heritage Education and Communication

In [1], the authors developed an educational game that utilizes both marker-based and location-based augmented reality called “The buildings speak about our city”. The game prompts students to explore tobacco warehouses in a city in Western Greece, which pose cultural, historical, and architectural interest. The game also encourages them to find out about the relationship of these tobacco warehouses with the city’s economic and cultural development.

The authors of [40] developed a location-aware AR application that superimposes a 3D model of the past state of a historical building in the real world and allows users to receive historical information in a multimedia form as they walk through the Venetian-style part of the city of Chania.

A location-based Augmented Reality system was developed in [37] to enhance archaeological heritage. The project consists of a mobile app to enhance points of interest (POIs) of the archaeological park of Castiglione di Paludi, Calabria, Italy.

Knossos AR [41] is an outdoor mobile augmented reality guide for the Unesco world heritage site of Knossos, the largest Bronze Age archaeological site on Crete (Greece), considered the oldest city in Europe.

In [42], the authors created BataviAR, a location-based AR application that provides informal learning to tourists visiting the historic site of Jakarta Old Town, and in [43], the authors created a real-time location-based mobile AR system for a cultural site in Mauritius that promotes cultural tourism and enhances the visiting experience.

Another relatively recent example of a location-based AR application for cultural heritage education is presented in [2]. This research paper deals with the development of a mobile location-based AR game that utilizes local history and cultural heritage in the game scenario. The game unfolds in the town of Kemijärvi, Northern Finland, in the 1920s, and its aim is to educate visitors and local residents about the town’s history and cultural heritage.

Location-based AR applications often follow gamification design principles and contain gamification elements [17]. The gamification elements that are frequently encountered in location-based AR applications are points, leaderboards, progress bars, ranks, and rewards [44,45,46,47,48]. Furthermore, gamification design principles include personalization, feedback, freedom of choice, failure, goals, challenges, and social engagement [44,48]. Storytelling and role-play can also be considered as a form of gamification. These applications can also contain digital avatars that can guide the users during the experience, and users can also take on certain roles during the game.

The Geist project was an early attempt to develop a storytelling AR application [3]. The system “GEIST” aims to provide information about historical facts and entertain the users by telling stories.

Another early example of an AR location-based game that utilizes storytelling to entertain and educate tourists is REXplorer [49]. REXplorer was specially designed for visitors to the town of Regensburg, Germany. During the AR guided tour, the tourists meet with digital characters through the application and, more specifically, spirits of historical figures that lived in the past. These digital encounters take place in historical buildings of the town. The spirits encourage the participants to complete certain tasks in specific locations within the city center. By performing these tasks, participants indirectly explore the town’s historic city center and learn history entertainingly.

A similar initiative to REXplorer is the SPIRIT research project [50]. Among the project’s aims, one was to develop a location-based Augmented Reality (AR) storytelling application where spirit figures tell stories that occurred during their lifetime at the Saalburg Roman Fort, which is nowadays an outdoor museum site.

Another example of a gamified storytelling AR location-based application is the “unlocking Porto” app [51]. During the location-based AR tour, the player is prompted to visit Porto’s main sights and play small games that appear in the application.

The authors of [52] present Viking Ghost Hunt (VGH), a location-aware adventure game based on a Gothic ghost story set in Viking Dublin. In this game, the users assume the role of a paranormal investigator. The investigator moves around in the city of Dublin, and his task is to hunt ghosts, collect evidence, and solve the mysteries of haunted Viking Dublin. The paper also evaluates the users’ engagement and immersion through qualitative methods.

Location-based AR applications can also be built for tours in interior spaces with the use of beacons as well as other techniques. For example, in [53], the researchers use beacons to develop a museum AR tour guide application that encompasses educational and entertainment functions.

## 3. The Doltso District and the Related Educational Program

Doltso is an old district of Kastoria, a city in the North-Western part of Greece. Kastoria has been inhabited since prehistoric times and experienced prosperity during the Byzantine and Post-Byzantine periods. With the Ottoman conquest, the city acquires a multi-ethnic character. Turks, Greeks, and Jews leave their mark on the city.

However, after the violent changes caused by—among others—the exchange of populations between Greece and Turkey and the Holocaust, the traditional form of the city is today only preserved in the old Greek districts, with the names Doltso and Apozari. These districts have numerous Byzantine and post-Byzantine churches, impressive mansions, and historical buildings. This historic core, inhabited since the middle Byzantine period, largely preserves the old city layout. Today restored mansions operate as museums, restaurants, cafés, and elegant guest houses. Several European-funded restoration and maintenance projects that have taken place recently contributed to preserving the city’s valuable cultural heritage.

The Center for Education for the Environment and Sustainability of Kastoria (previously called the Center of Environmental Education of Kastoria), adopting the modern global directions for the promotion of sustainable development, designed and implemented the educational program “Doltso: A traditional district of Kastoria over the passage of time” in 2018. The program focused on the conservation and sustainable management of cultural heritage and was aimed at primary and secondary education students. The duration of the program was initially four teaching hours, and the program’s experiential activities took place in the historical center of the city of Kastoria, the “Doltso” district.

In 2022, the program was transformed to include AR technology to enhance the experience by gamifying the learning process. More specifically, a location-aware application has been developed that guides the students through the district and cobblestone streets and stairways, prompting them to observe the district monuments (mansions, byzantine and post byzantine churches, etc.) and to obtain information through the digital application. Through the AR tour, the students are also prompted to answer relevant questions regarding the monuments and gather points when these questions are answered correctly.

The application is a joint effort between the DMSClab (Digital Media and Strategic Communication Lab) of the Communication and Digital Media Department, University of Western Macedonia, Greece, and the Center for Education for the Environment and Sustainability of Kastoria. These two entities are in close collaboration to implement research and education projects related to new technologies in education.

Seeking to exploit the advantages offered by location-aware AR technologies, the augmented reality digital application “Doltso: A traditional district of Kastoria in the passage of time” was designed with the following objectives in mind:

- To provide an alternative method that will mobilize the interest of the students and strengthen their active participation during the fieldwork of the educational program.
- To strengthen the experiential dimension of the educational activity through the capabilities and incentives provided by the continuous interaction of students with real elements of the physical environment and the digital elements of the AR mobile application.
- To evoke positive feelings usually present when students participate in games that encourage cooperation and are supported using their favorite means of entertainment and communication devices (smartphones and tablets).
- To utilize and further develop the students’ digital literacy.
- To provide students with attractive means of obtaining information about a historical area of the city of Kastoria with significant cultural value. This learning resource could also be used anytime again in the future. More specifically, the students can visit the area alone or with their family or friends and experience the AR game at their own time and pace.

A virtual reality (VR) tour was also developed using Google Earth in order to be used in the classroom or at home after the AR educational tour. The VR tour that guides the users through the same places as the AR tour is optional and is provided as a mean for refreshing and consolidating the knowledge acquired through the AR tour.

## 4. Materials and Methods

The development of the AR application went through the following stages: requirement analysis, application design, prototype implementation, testing, application finalization, usage, and evaluation (as described in Section 5). In the first two stages, certain considerations were needed to be made. These included the target audience, the authoring tools that would be used for creating the application, and considerations regarding the application scenario.

During the requirements analysis phase, the literature was searched in order to identify specific requirements of location-based AR applications for cultural heritage education as well as good practices. Ideas were also exchanged between the research team and the educators of the Center for Education for the Environment and Sustainability of Kastoria. The literature was also investigated in order to find the appropriate software tool for the development of the application keeping in mind that one of the study’s objectives was to create an application that would inspire educators wishing to employ location-based AR in their cultural heritage education projects, providing them with ideas on how to proceed on such a mission. As it will be described in the next section, a thorough analysis of various software tools was carried out in previous studies to decide on the appropriate software tool.

In this study, Taleblazer was chosen for the application creation, a reliable software tool developed by MIT with many capabilities for building mobile location-based AR applications that educators can use without advanced programming expertise. Image editing tools were also used in order to edit the images used in the application. The sources for the information included in the application were obtained from books and credible web resources.

Initially, a prototype application was developed. The prototype included a subset of the locations as well as content (information, pictures, and questions) associated with these locations. The prototype was tested by members of the research team, the educators of the Center for Education for the Environment and Sustainability of Kastoria, and a small sample of students. The testing phase revealed some problems that led to the re-design of some application aspects and changes in the Taleblazer configuration settings.

Finally, more content was added to the application, and the final version was obtained after a last round of testing to eliminate errors.

A sample of 309 school students was used to evaluate the application. For the evaluation, a questionnaire was created using latent variables that are considered suitable for location-based applications: challenge, educational usefulness, collaboration, and intention to reuse. These constructs and their items (questions) were mainly derived from evaluation models from the existing literature. The constructs are presented in Section 7, and the whole questionnaire is presented in Appendix A.

Descriptive statistics (mean, standard deviation, standardized coefficients) were obtained using SPSS, and structural equation modeling analysis was carried out using the software AMOS (version 21.0). This analysis aimed to obtain a model representing how the constructs are causally related.

Furthermore, as mentioned in the previous sections, a Google Earth VR tour was also created in order to be used after the augmented reality experience (in class time or at home) to consolidate the acquired knowledge. The creation of this project has also gone through similar development stages as the AR application, contains the same locations and follows a similar scenario. HTML and Javascript libraries were also used to enhance the interactivity of the project. Details of the Google Earth VR tour are presented in Section 6.

## 5. Creating the Location-Based AR Application “Doltso: A Traditional District of Kastoria in the Passage of Time”

**Figure 1:**
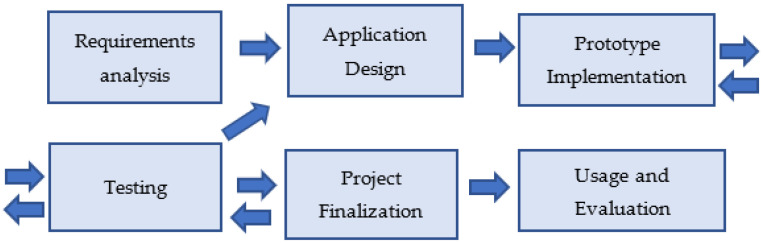
Project stages.

The creation of the AR application followed typical steps in a software development life cycle (Figure 1).

In the requirements analysis phase, specific considerations and decisions needed to be made, such as determining the application’s target audience and the software tools that would be used for implementing the application. In the application design phase, the user interface, the application scenario, and the game mechanics were designed. It was a design decision to introduce different roles, gamification elements (e.g., points, awards), and elements that would also keep the focus on the real world and the aspects of cultural heritage interest (questions that require the observation of the physical surroundings). Members of the research team determined the application content in close collaboration with the educators of the Center for Education for the Environment and Sustainability of Kastoria.

A prototype implementation then took place. The prototype, which included only a subset of the final content, was tested by the experienced educators of the Center for Education for the Environment and Sustainability of Kastoria, two IT experts of the research team, and a small sample of K-12 and higher education students. The testing phase revealed software errors (bugs) and aspects of the application that needed re-design and tuning (e.g., game mechanics, content, configuration parameters).

Finally, after dealing with the issues revealed in the testing phase of the prototype, the final version of the project was created by adding all the necessary information. The final version was also tested to eliminate any errors. Furthermore, a webpage was created to host and promote the created application. Important instructions were also included on this web page for the application installation and operation.

The application was experienced by a large number of students and evaluated by a sample of 309 students who took part in the educational program. The evaluation questionnaire was constructed by utilizing the existing literature. Some details of the production stages are given in the next sub-sections.

### 5.1. Requirement Analysis Considerations

Regarding the target audience, although the application will be experienced mostly by students participating in the educational program, it was an initial decision that it would also be available to every citizen of Kastoria and every tourist interested in learning about the history and culture of the city. With this goal in mind, a significant amount of information would have to be gathered regarding the old city monuments in the design phase and incorporated into the application.

Another consideration was the tool that would be used to develop such an application. By examining the related scientific literature, it became evident that in most studies and initiatives, AR applications are created with tools that require advanced programming expertise (e.g., Unity, Vuforia, ARkit, and ARcore are the most popular solutions). Software authoring tools that require little or no programming knowledge (e.g., ARIS, Taleblazer) and thus are suitable for most educators [17], are only utilized in few studies.

**Figure 2:**
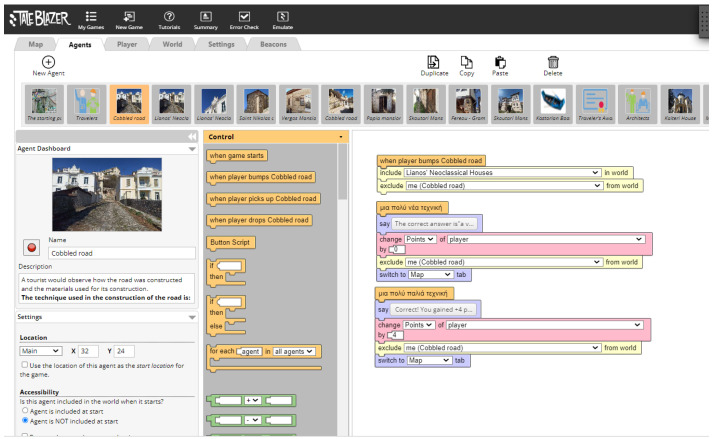
Taleblazer visual blocked-based programming environment.

As mentioned, this initiative aimed to make an application that would inspire educators and provide them with ideas on transforming educational cultural heritage tours into AR experiences. Although most projects dealing with location-based AR utilize tools that require advanced programming knowledge, in our case, tools that are accessible and user-friendly to a broader audience were needed. In this category, there are tools that do not require programming (e.g., Metaverse Studio, Web AR, Action Bound) and tools that are suitable for novice programmers. Taleblazer is such a tool. TaleBlazer was developed by the “Scheller Teacher Education Program (STEP)” of the Massachusetts Institute of Technology (Massachusetts Institute of Technology, MIT). Taleblazer has a visual block-based programming environment similar to Scratch, another popular product of MIT (Figure 2).

In previous studies [54,55], we explored the capabilities of various tools. More specifically, in [54], we conducted a full-fledged comparative analysis between Taleblazer and Metaverse studio, another popular tool used for building location-based AR experiences. In this analysis, the affordances of the two platforms were explored by examining the developer environment, the user interface of third-party applications, and the documentation of each platform and by developing prototype applications with both tools. In [55], we concentrated on finding the appropriate tool for creating AR applications for cultural heritage education. The studies revealed that although Metaverse studio has a broader range of multimedia elements that can be incorporated into the AR experience, Taleblazer has more advantages for building cultural heritage applications which are briefly presented below:

- Taleblazer utilizes more detailed maps (Google Map API vs. Open Map) that aid the navigation through city streets.
- Taleblazer provides the ability to use custom maps besides the Google dynamic map. This means that creators can create their own maps using image editing and graphic design tools, and applications can be experienced on these custom maps as long as they are matched appropriately with the physical locations. Using custom maps, the creator can include details of the physical environment that cannot be depicted in Google Maps, such as nature trails and narrow city paths. The creator can also include imaginary content. Furthermore, by using custom maps, applications can be experienced without an internet connection. Although many students (and people in general) have cellular data on their mobile phones through their subscriptions, many students do not have such an option. For this reason, it was decided to use custom maps for the Doltso application. Another reason for using custom maps in our application was that the GPS signal seemed to be more stable with this option.
- Furthermore, custom maps can be used to build applications for interior spaces, and instructions on how to achieve this are provided by the Taleblazer documentation.
- Taleblazer is efficient at supporting role-playing games. The developer can easily create different roles, and various scenarios can be built around these roles. This feature is handy for designing storytelling experiences.
- The Taleblazer environment allows the configuration of the “bump” or “tap to bump” settings. Users are said to “bump” into a GPS location when they get in a certain proximity from the location. Digital content is then activated automatically. Digital content can also be activated when the GPS location icons on the map are tapped (tap to bump). The proximity values can be configured in the settings table. In other AR platforms, such as Metaverse studio, this setting is fixed and cannot be modified (e.g., 30 m radius). This feature that is present in Taleblazer can be valuable in many cases. For example, the creator may want the player to get very close to a physical location to observe things that are not visible from a distance, such as a small statue or signs on walls and doors. This is also the case with the application of Doltso where the user is prompted to observe various things from proximity (e.g., signs on the walls of churches and mansions, architectural aspects, etc.)
- Taleblazer can support games or tours where the points of interest appear in sequential order. In Taleblazer, the GPS locations (or Agents in the Taleblazer terminology) can be made visible or invisible on demand, supporting the development of such games.
- Taleblazer games can also be tested and experienced away from the physical world game area. A specific setting in the programming environment called “tap to visit” can enable or disable this option. This option is disabled by default and enabled only by issuing a correct password defined by the application developer. This option can be enabled when testing the AR applications (simulation mode) and in cases where the creators want to distribute the application to everyone who cannot visit the location where the game unfolds. This feature is not present in Metaverse studio, and this is a major disadvantage since an application cannot be tested or experienced off-site.

### 5.2. Application Design Aspects

A consideration that had to be made in the design phase was the application scenario. Mads Haahr [56] claims that offering immersion during gameplay without losing the sense of presence within physical space is a major challenge for location-based AR games. For example, Pokemon GO is an experience that immerses people into the gameplay without keeping their attention to their surroundings in the real world.

While this may be acceptable for a non-cultural game experience, it is unsuitable for site-specific cultural heritage games where the site itself plays a crucial role in the experience. Mechanics such as those adopted in Pokemon GO and Ingress are generally not suitable for location-based cultural heritage games since they sacrifice presence in the physical space for immersion into the game world.

Keeping this in mind: an objective of the application scenario would be to keep the focus of the users both on the digital content of the application and also on their immediate surroundings. To achieve this, questions that can be answered by observation were introduced throughout the game.

Roles, points, and awards were also adopted in order to gamify the experience. The users through the roles of the tourist, architect, local resident, and active citizen are motivated to observe, think, learn, and express their views about different aspects concerning the monuments.

In the initial screen of the application, the users are prompted to read the application instructions in order to obtain crucial information on how to navigate the application. Then the users take on four consecutive roles and answer multiple-choice questions that are specific to the roles. First, the users take on the role of the tourist and visit 11 locations while they are prompted to answer an equal number of questions. If the answer is correct, the user receives points (four or two in cases where an answer is partially correct). The points can be seen at any time through the application menu. After visiting all the locations as a tourist, the users receive the tourist award. It was a design decision to give the awards if all the required locations were visited regardless of the achieved score.

Then this process is repeated for the rest of the roles. The architect (five locations), local resident (five locations), and active citizen (four locations). Awards are again given when the tasks are completed. The objectives of each role are described later in this section.

**Figure 3:**
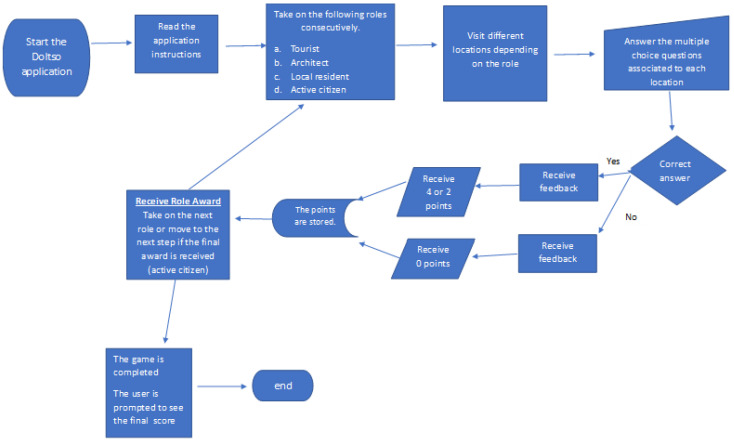
Flow chart diagram of the Doltso application.

The application design is depicted in the flow diagram below (Figure 3):

As a tourist, the users are motivated to achieve the following things:

- Learn about the monuments that exist in the area (e.g., mansions, churches, cobbled streets).
- Conclude about the monuments’ time of construction, the social and economic conditions of that time, and the peoples’ way of life and daily activities throughout different time periods.
- Recognize the decisive contribution of the lake ecosystem to the social and economic development of the city and the role it played in shaping the cultural identity.

As an architect, the users are motivated to achieve the following things:

- Learn about the materials used in the constructions and the techniques used.
- Observe and learn about the architectural features of buildings from earlier eras, the purpose that these features serve, and the design patterns of public infrastructure.

As a resident of the area, the users are prompted to achieve the following things:

- Learn about the current uses of the monuments (e.g., mansions) and how these help in the preservation of the monuments.

As an active citizen the users are motivated to achieve the following things:

- Reflect and express their views on the collective and individual responsibility regarding the conservation of monument buildings.
- Propose directions and practices for the monument’s harmonious and functional integration into the modern residential landscape.
- Evaluate the prospect of the overall regeneration of the historic city center.

### 5.3. Implementation

**Figure 4:**
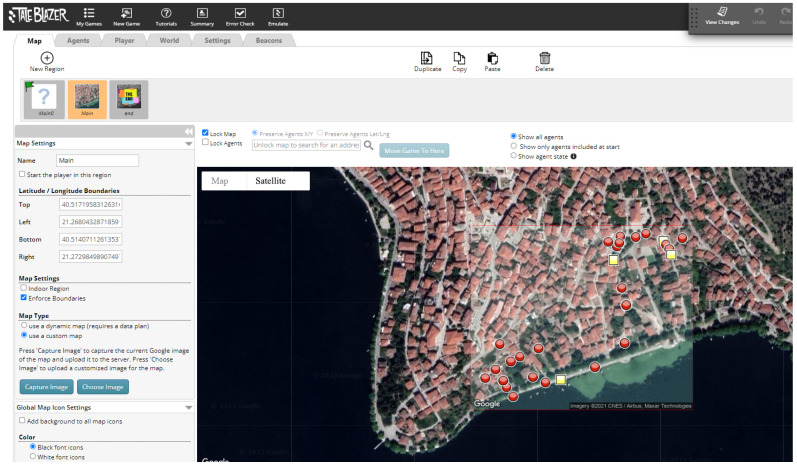
Setting the region of the game in Taleblazer and inserting Agents (red circles and yellow boxes).

The basic elements of the Taleblazer programming environment are “Regions” and “Agents”. Regions are the physical areas on the map where the game (or tour) will evolve. Using a selection tool, the designer can determine the game’s region on a digital map. After the region is set then, “Agents” can be introduced. Agents are multimedia elements associated with a GPS location and activated when a learner “bumps” into this location (Figure 4). This content can be in the form of images, multiple-choice questions, sounds, narration, and video.

A unique code is automatically created for every game app designed in TaleBlazer.

To experience the application, the user would have to download Taleblazer from “Google Play” or “App Store” for Android or IOS operating systems, respectively, and then insert the unique code in the “Game Code” tab to install the game.

In the previous section, it was mentioned that we decided to use custom maps so that an internet connection is not required, making the application also accessible to students and town visitors who do not have cellular data activated on their phones.

As already mentioned, before reaching the final version, a prototype application was developed and tested. The prototype contained a subset of the final content (locations, information, and questions). The testing phase of the prototype revealed software errors (bugs) and aspects that needed re-design. Software errors were dealt with, and the following aspects were re-designed:

- Application information and questions. Incomprehensive directives and text in questions were re-written.
- The mechanism for collecting points and awards. It was decided to issue points when questions were answered correctly and awards when the users progress in the game after visiting a number of locations. This decision was taken in order to keep students motivated and avoid discouragement when questions are not answered correctly.
- Taleblazer configuration parameters. Certain parameters were tuned in order for the digital content to be activated at the appropriate GPS locations (e.g., the bump and tap-to-bump options as described in detail in the previous section).
- Application instructions. A problem that arose during testing was the loss of the GPS signal in some devices. This problem occurred while walking in the narrow streets of the Doltso district, where the GPS signal might have been blocked by the nearby buildings. Instructions on what to do in this case were included in the initial screens of the application.

After the testing of the prototype, more content was added to the application in order to create the final version. The final version was also tested to eliminate any errors that may have resulted from the addition of the new content. Today, this final version is used in the educational program of the Environment and Sustainability Education Centre of Kastoria and is also available to everyone interested in learning about the history and monuments of the Doltso district.

**Figure 5:**
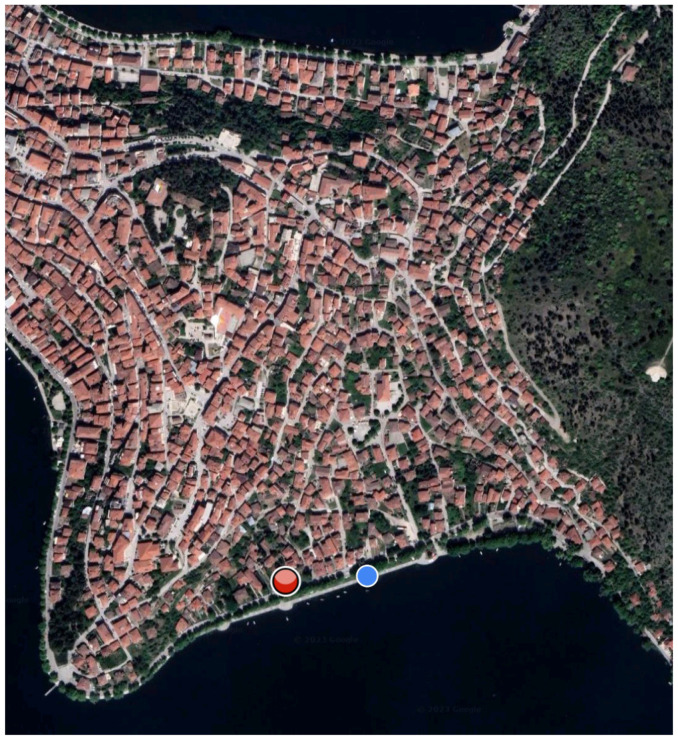
Application map where the position of the user is indicated with the blue dot and the position of the agent (point of interest) with a red dot.

The students taking part in the program use the digital application and follow a route through the area of Doltso, which includes twenty-five observation points/stations. The duration of the tour is about 1.5 h. The application guides the students so they can reach points of interest. The student’s position is constantly being tracked by the GPS sensor of the mobile device, and a blue dot depicts it. On the other hand, the points of interest appear as red or yellow dots (Figure 5). The application was designed so that the locations are visited in sequential order. Thus, only the next location to be visited appears on the digital map. 

**Figure 6:**
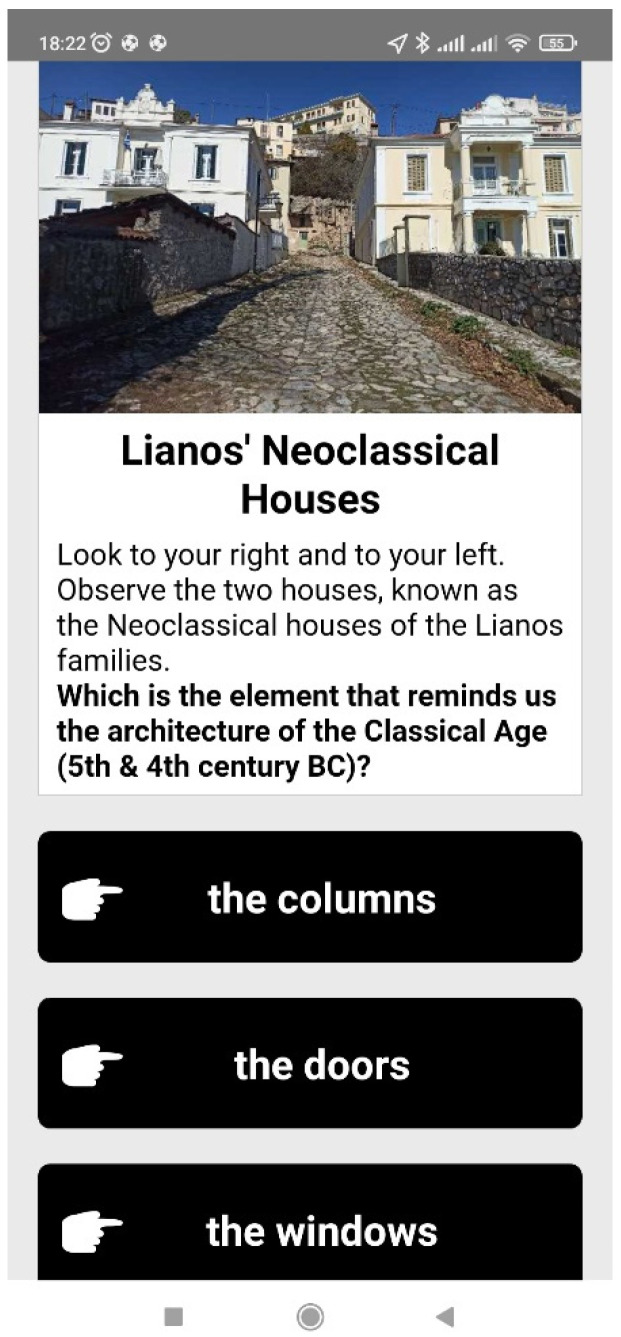
Question regarding the architecture of a house found in a historical district.

**Figure 7:**
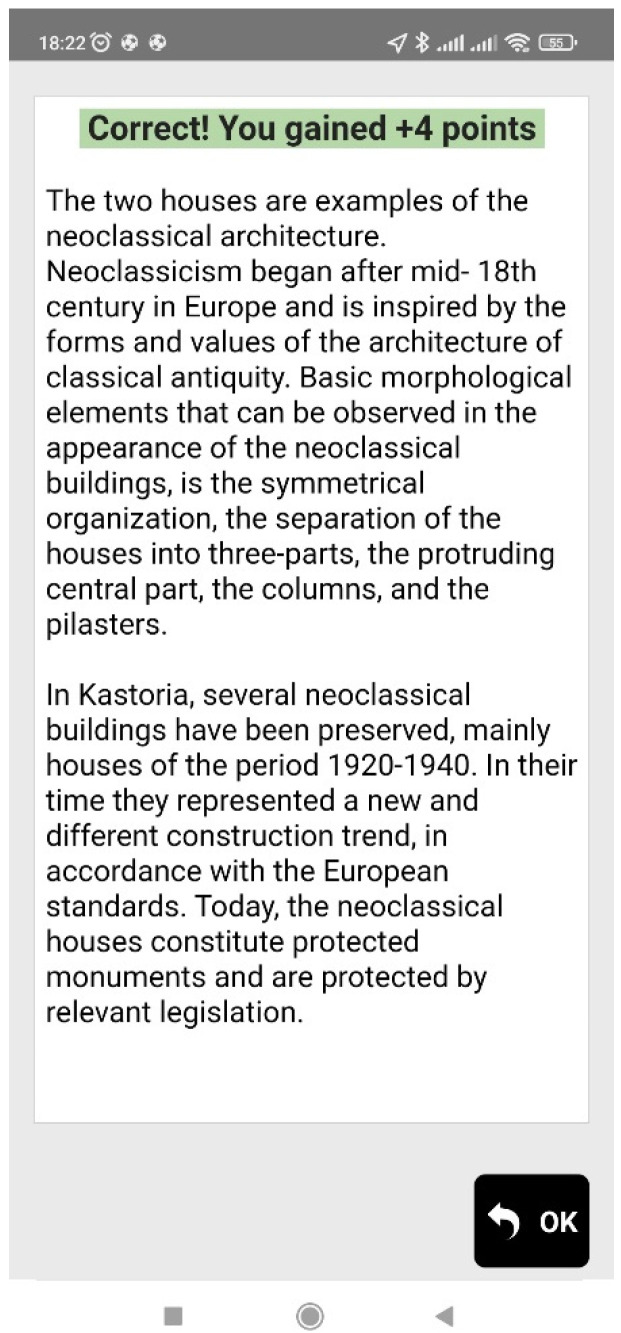
Information received as feedback.

**Figure 8:**
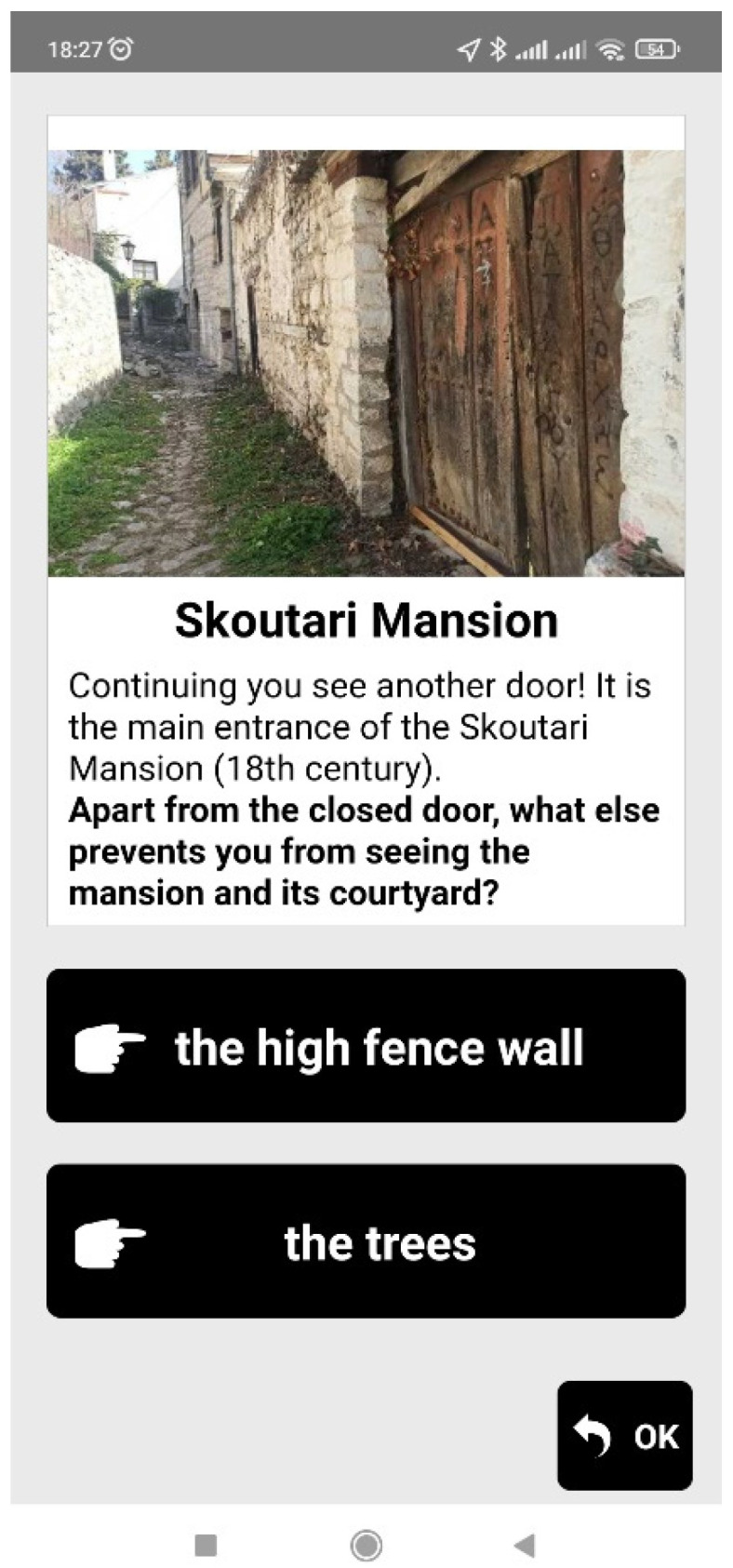
Question regarding aspects of a mansion construction.

**Figure 9:**
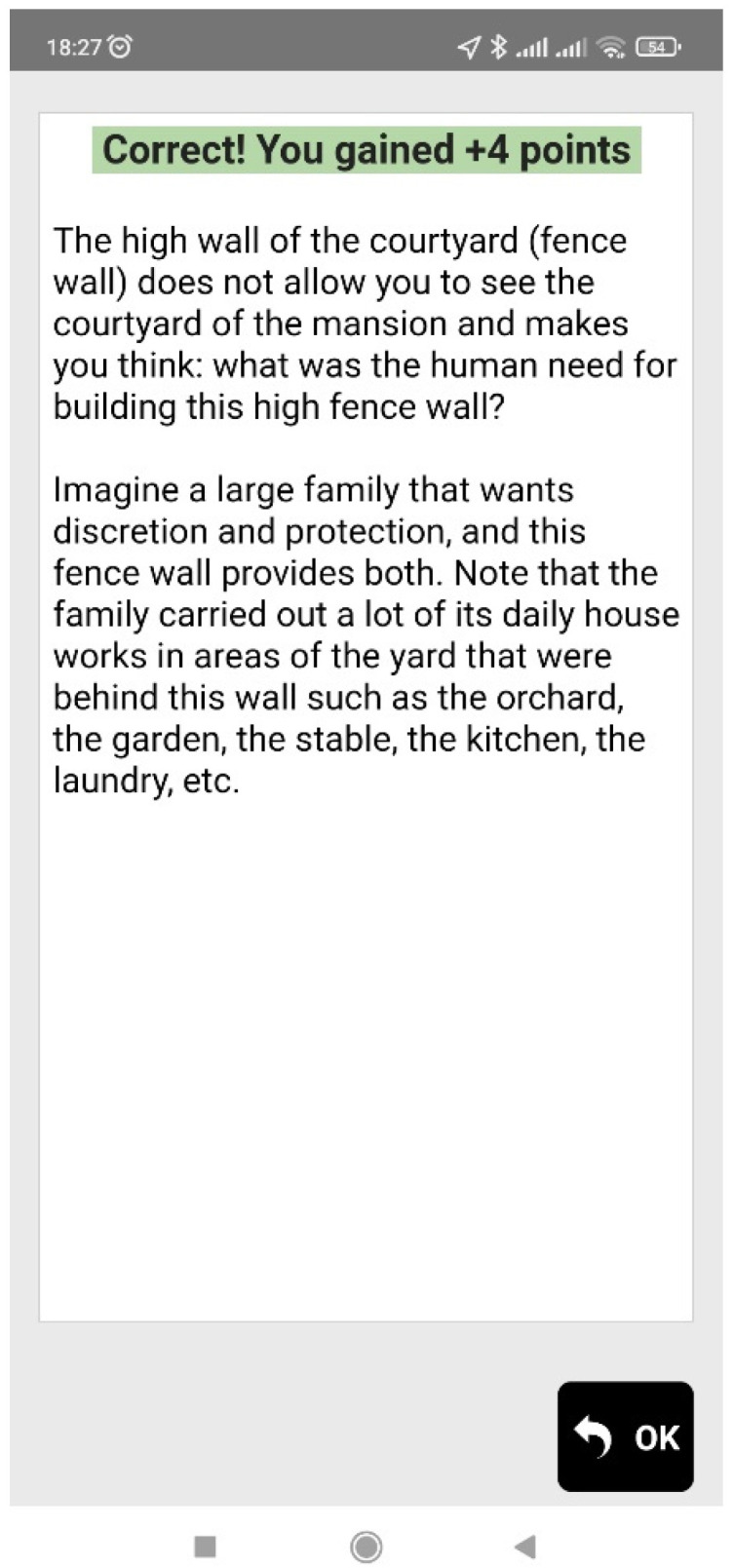
Information received as feedback.

When the user reaches a point of interest (that is, when the blue dot of the user gets close to the red or yellow dot of the Agent), informative multimedia content in the form of text and an image appears on the screen of the mobile device, and the users are also often prompted to observe their surroundings (e.g., a building, a backyard, church, etc.) in order to answer a multiple-choice question. Feedback is then given to the users, informing them if the answer is correct or not, and more information regarding the question topic is provided. Figure 6, Figure 7, Figure 8 and Figure 9 are examples of two questions and feedback screens of the application. By answering the questions, the users gather points (as can be seen in Figure 7 and Figure 9) and receive digital awards in the end.

**Figure 10:**
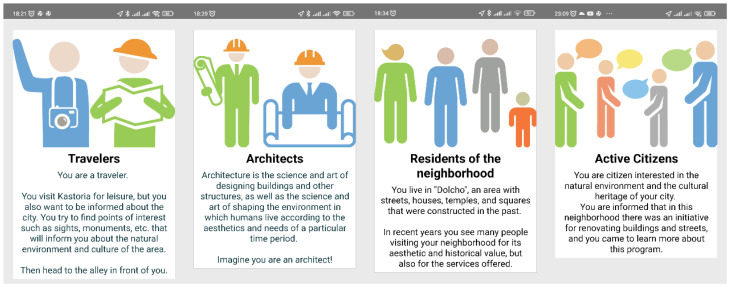
Screens of the roles undertaken by the users during gameplay.

During the AR tour, the users, as mentioned in the application design section, successively adopt four roles: the roles of the tourist, the architect, the local resident, and the active citizen (Figure 10). Through these roles, an attempt is made to highlight the aesthetic, historical, architectural, and economic value of the area’s monuments and how these monuments are currently integrated into modern living.

**Figure 11:**
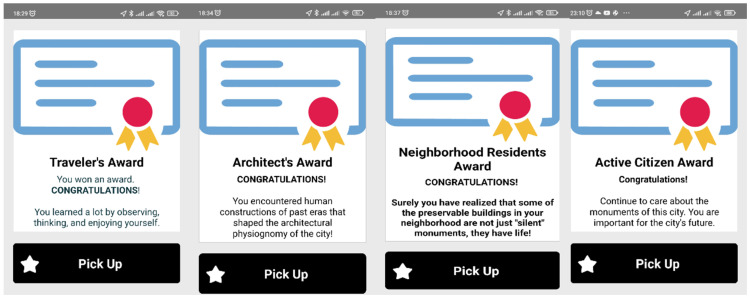
Screens of the Awards given to the users at various points in the AR tour.

Digital awards are provided on successful completion of the tour (Figure 11).

The digital application “Doltso: A Traditional District of Kastoria in Passage of Time” is available in two languages, Greek and English. The codes gmfavxg and gafatus are needed to download these versions, respectively. Detailed installation instructions, presentation material, demonstration videos, and related posters can be found on the website of the Environment and Sustainability Education Centre of Kastoria (https://kpekastor.kas.sch.gr/taleblazer accessed on 17 February 2023).

## 6. Creating a Virtual Reality Tour with Google Earth Projects

A Google Earth project was also developed in order to be used optionally in a classroom or at home after the AR educational tour. The aim of the VR tour was to consolidate the knowledge acquired by the AR application.

The tools provided by Google Earth (https://earth.google.com/web/ accessed on 17 February 2023) allow educators to create and share digital tours and storytelling experiences related to places in the world. Educators can create a project by adding points, drawing placemarks, lines, and shapes and associating rich contextual information with locations in the world (text, links, images, videos, 3D views, and street view). The content is then organized in a table of contents, and the places can be visited in a narrative flow. Educators can share their projects with their students and collaborate with other educators to build these experiences.

In presentation mode, the viewers will fly from one place to the next, following the project’s narrative, immersing in the journey through Google Earth’s imagery and the custom content provided.

**Figure 12:**
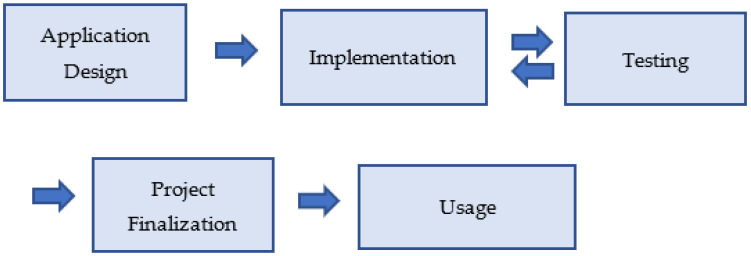
Development stages of the Google Earth VR tour.

The steps followed for the implementation of the Google Earth VR tour are depicted in Figure 12:

The places in the Google Earth project were the same as those in the augmented reality tour created by Taleblazer. Hence, the application design contained the same roles (tourist, architect, local resident, and active citizen) and the same locations. More specifically, the users can visit the places virtually using street view and/or photo images and complete similar tasks as the real-world AR tour. More specifically, the students can observe the monuments through the street view option or the photos embedded in the application and answer related questions. Feedback is then provided to the users, informing them about the right answer and providing them with detailed information. The only difference in the Google Earth project was the absence of points given as a reward. Points cannot be stored in a database through the standard Google Earth functionality.

In order to create the virtual reality (VR) tour, all the affordances of Google Earth were utilized. Furthermore, HTML, CSS and Javascript coding (e.g., HTML custom info boxes, buttons, and links) were used to in order to insert questions and answers and enhance the interactivity of the Google Earth project.

The following resources were used during the implementation of the project: Google Earth on Web: https://earth.google.com/web/ accessed on 17 February 2023, W3Schools: https://www.w3schools.com/tags/ accessed on 17 February 2023, Visual Studio Code: https://code.visualstudio.com/ accessed on 17 February 2023, GitHub for this training: https://github.com/geteach/htmlEarthTrain3 accessed on 17 February 2023, and Material Design Lite: https://getmdl.io/ accessed on 17 February 2023.

The tour is only available in Greek at the moment from http://tiny.cc/rujluz accessed on 17 February 2023.

**Figure 13:**
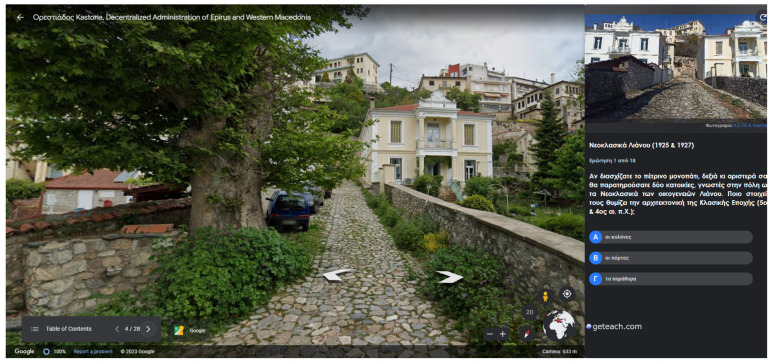
Street view of the Google Earth project. A multiple-choice question regarding a city’s monument appears on the right side of the screen. The blue circles indicate the 3 questions.

**Figure 14:**
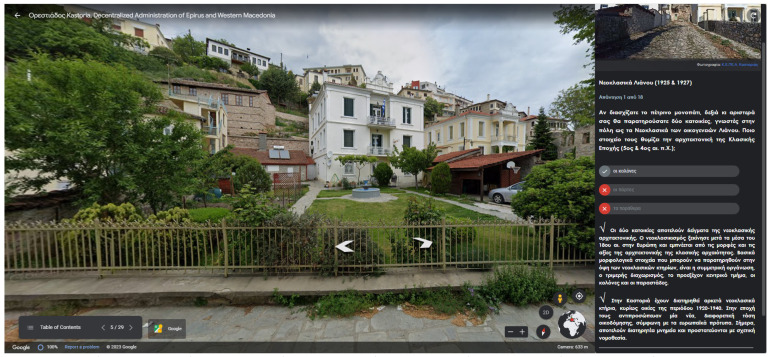
Feedback given to the user after answering a multiple-choice question. The wrong answers are indicated by the red circles. Information is given as feedback below the answers.

Two screens from the Google Earth project are given in Figure 13 and Figure 14. In Figure 13, the users are prompted to observe a house with neoclassical architecture and answer a multiple-choice question regarding the house’s architecture. In Figure 14, feedback is given to the user after answering a multiple-choice question. The feedback contains the right answer and detailed information.

## 7. Evaluation

Up to date, around 1100 students have participated in the program and experienced the AR tour.

In order to evaluate the student experience, a questionnaire was constructed and delivered to a sample of 309 students shortly after experiencing the AR tour. The self-administered questionnaire was comprised of the following constructs: challenge, outdoor activity, ease of use, usefulness (knowledge), interaction, and intention to reuse the application or similar application.

The questions belonging to the above constructs were mainly derived from the existing literature and validated questionnaires and adapted to the purposes of the study.

The questionnaire consisted of 19 Likert-scale questions belonging to 6 constructs (see Appendix A).

The constructs were the following:

- Challenge: the feeling players get when they proceed in the game (proud, satisfied, excited, etc.).
- Outdoor activity: the motivation triggered by the application to engage in an outdoor activity.
- Ease of use.
- Perceived usefulness/knowledge: improving users’ knowledge about the cultural aspects of the Doltso district.
- Interaction and collaboration amongst co-players.
- Intention to reuse the same application or to experience similar applications for other places of cultural heritage interest.

More specifically, questions measuring “Challenge” were derived from [57] and assessed the extent to which the players felt pride, feelings of reward, and excitement when using the application. Outdoor activity was measured through questions derived from [58]. These questions evaluated whether the application motivated players to go out and explore new places as well as to meet their friends outdoors.

Knowledge (perceived educational usefulness), ease of use, and intention to reuse were derived from [59]. Perceived educational usefulness was related to the improvement of players’ knowledge about the cultural heritage of the city, while the intention to reuse measured the players’ intention to play and experience the application or similar applications again in the future. Interaction & collaboration were measured using two items constructed for the purposes of this study in order to assess the level of cooperation and collaboration among students while experiencing the AR tour.

## 8. Results and Discussion

**Figure 15:**
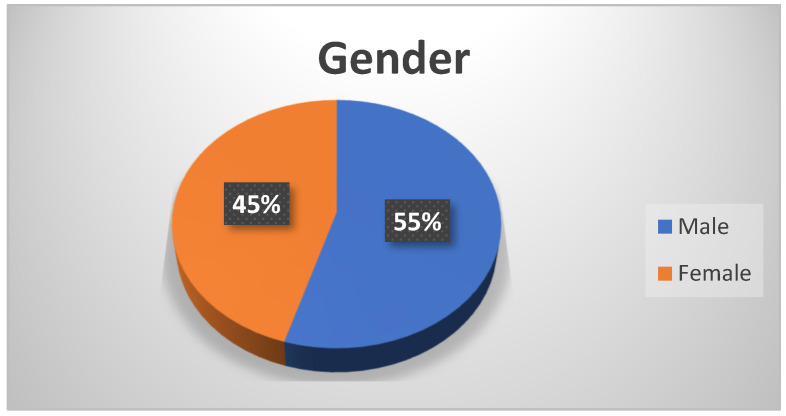
Gender of the participants.

From the sample of 309 students, 169 were boys, and 140 were girls (Figure 15).

**Figure 16:**
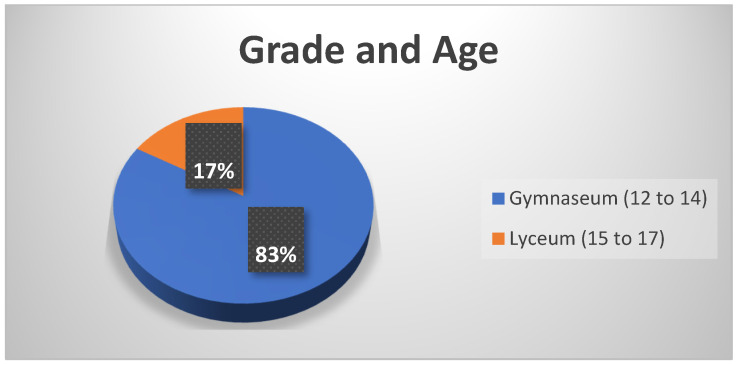
Educational Level.

The students were mainly from the first three grades of high school. More specifically, 258 were from grades A, B, and C (89, 82, and 87, respectively). The age range of these students was between 12 and 14. The rest 51 students were in grades D and E of high school (44 and 7, respectively). In Greece, the term Lyceum is used for the last three grades of secondary education. The age range of these students was 15 to 17 (Figure 16).

**Figure 17:**
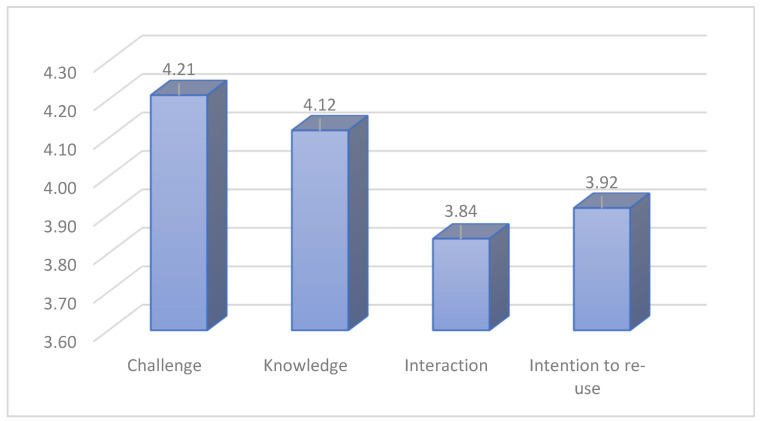
Mean scores of constructs.

To analyze the data and achieve the research objectives, a two-step procedure was followed. First, the five constructs were evaluated for their internal consistency via SPSS 21.0 using Cronbach’s alpha. Based on the results, the constructs of challenge (a = 0.828), usefulness (knowledge) (a = 0.794), interaction (a = 0.745), and intention to reuse (a = 0.721) showed satisfactory internal reliability as they exceeded the 0.70 threshold. On the other hand, the constructs of outdoor activity (a = 0.650) and the ease of use (a = 0.036) were dropped from further analysis since they exhibited low internal reliability. Figure 17 shows the mean scores of the average summated scales that were developed for the constructs of challenge, knowledge, interaction, and intention to reuse.

Descriptive statistical values for every item of the four categories are given in Table 1.

Based on the results, players felt intense feelings of challenge (M = 4.21, SD = 0.81) and perceived the application as highly useful in terms of cultural heritage knowledge gains (M = 4.12, SD = 0.79). Moderate to high levels of interaction (M = 3.83, SD = 0.93) and intentions to reuse the application were exhibited by the participants of the survey (M = 3.92, SD = 0.88). As one can see from Table 1, however, although the intention to play the same game is moderate, the intention to play a similar game with new content for another district of the city is high.

### 8.1. Measurement Model

**Figure 18:**
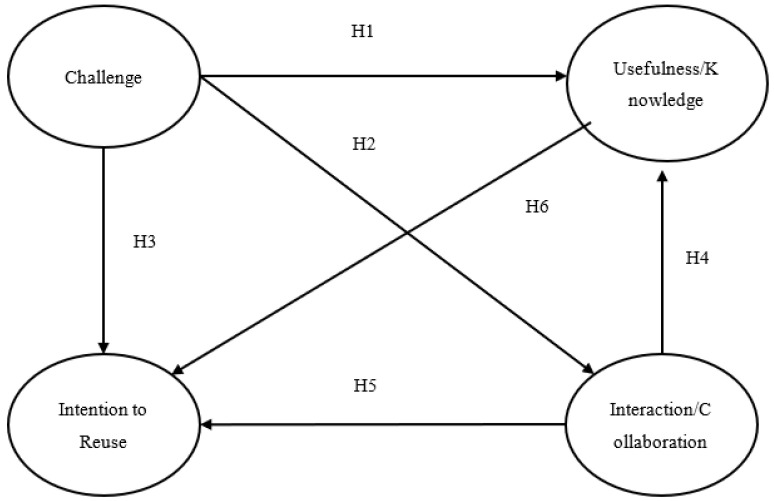
Conceptual Framework.

To test the relationships of the conceptual framework of the study (Figure 18), structural equation modeling was performed by using Amos 21.0.

First, we tested the measurement model and calculated the goodness-of-fit measures as well as the construct, composite, and discriminant validity of the latent constructs used. The measurement model consisted of four latent variables (challenge, knowledge, interaction, and intention to reuse) and ten observed variables. The goodness-of-fit measures suggested a good fit for the model (χ2 = 72.66, *p* = 0.000, χ2/df = 2.51, Comparative Fit Index (CFI) = 0.967, Goodness of Fit Index (GFI) = 0.955, Tucker–Lewis’s index [TLI] = 0.949) since the GFI, CFI, and TLI values exceeded the 0.90 criterion proposed by Byrne in 2010 [60]. The root mean square error of approximation (RMSEA) value was 0.07, thus smaller than the 0.08 threshold, indicating an adequate fit [61].

The four scales were evaluated for their convergent and discriminant validity. The standardized factor loadings of the items of the four scales were all significant (critical ratios >1.96, *p* = 0.000) and ranged from 0.577 to 0.857, exceeding the 0.50 cut-off value). The average variance extracted (AVE) for all constructs ranged from 0.532 to 0.65, exceeding Fornell and Larcker’s (1981) [62] cut-off value of 0.50. Furthermore, composite reliabilities of all constructs exceeded the accepted 0.70 value [63]. Thus, the constructs of challenge, usefulness (knowledge), interaction, and reuse intention showed acceptable levels of convergent validity. Moreover, the discriminant validity of the scales was satisfactory since the AVE of each scale was larger than the square of the correlation between the examined construct and the rest of the constructs (Table 2).

A structural equation analysis was performed to test the model’s relationships. The goodness-of-fit measures of the structural equation model indicated a satisfactory fit (χ2 = 72,6, *p* = 0.000, χ2/df = 2.51, CFI = 0.967, GFI = 0.955, IFI = 0.967, TLI = 0.949). Moreover, the RMSEA value was 0.07. Thus, the study’s model showed an acceptable fit to the data. The hypothesized relationships were then tested by examining the standardized estimates of the path coefficients (Table 3).

Based on the results (Table 3), the construct of challenge was a significant (*p* < 0.05) and positive predictor of the player’s perceived usefulness (knowledge) (b = 0.459, sig = 0.000) and interaction levels (b = 0.645, sig = 0.000). On the contrary, challenge did not significantly influence (*p* > 0.05) players’ re-usage intentions in a direct manner (b = 0.060, sig = 0.626). Thus, hypotheses H1 and H2 are accepted, while H3 is rejected. Interaction and collaboration with other players had a significant positive impact on players’ perceived usefulness/knowledge (b = 0.374, sig = 0.000). The impact of interaction/collaboration on players’ re-usage intention was non-significant (b = 0.105, sig = 0.285). Hence, H4 is accepted, while H5 is rejected. In addition, perceived usefulness/knowledge had a positive impact on users’ intention to reuse the application (b = 0.624, sig = 0.000) in the future, supporting H6.

The equations for the structural model are given in Appendix B.

Furthermore, we tested the indirect effects of the constructs using the bootstrap method on AMOS 21.0. Results are presented in Table 4. Based on the results, challenge had significant indirect effects on usefulness (knowledge) (b = 0.209, sig = 0.004) through the mediation of interaction. Moreover, challenge also had indirect effects on re-usage intentions (b = 0.574, sig = 0.003). The effects are mediated by the constructs of usefulness/knowledge and interaction. Moreover, usefulness/knowledge was also found to be a significant mediator of the relationship between interaction and re-usage intentions (b = 0.247, sig = 0.004). Thus, it can be argued that although challenge and interaction did not have direct effects on re-usage intentions, the construct of educational usefulness/knowledge can be regarded as an important mediator of the above relationships.

### 8.2. Limitations and Recommendations for Future Research

The descriptive statistical analysis and the structural equation modeling analysis results apply to the specific application and the specific location (Doltso district), as well as the specific educational settings (K-12 students participating in an educational program), and cannot be generalized. In order to obtain a broader perspective on how AR location-based applications designed for cultural heritage education using Taleblazer affect factors such as challenge, knowledge, collaboration, and intention to reuse, more similar evaluations will be needed in order to compare the results and provide more generalized insights. The research team of this paper has already designed similar applications for other districts of the city of Kastoria that have cultural heritage value, archeological sites, as well as tours of environmental interest and pure touristic interest. These applications are currently being evaluated by a large number of students that take place in the educational programs of the Center for Education for the Environment and Sustainability of Kastoria as well as visitors of the area.

Another limitation of the study concerns the VR Google Earth tour that is presented in this study. Google Earth tour was utilized to create an interactive project for consolidating the knowledge acquired in the augmented reality tour, but in this study, only the augmented reality application was evaluated since the VR tour was optional for the teachers and students to use in classroom time or at home. In the future, we plan to evaluate the whole experience (AR application and Google Earth VR tour) in terms of knowledge gains and engagement.

From the analysis, it became evident that the collaboration and interaction amongst students scored lower than other factors, such as challenge and educational usefulness/knowledge. Taking this in mind, the research team plans to explore new ways to encourage collaboration and interaction and design a new application version that enhances this dimension. One possible way of doing this is by assigning groups and instructing students that belong to the same group to take on different roles. Each student will collect points by answering questions specific to the role assigned to them, and these points can be added up in the end to make the group total. The students will be free to collaborate within their group. The teacher, in the end, will acknowledge the winning group. This is an example of a procedure that could enhance student collaboration and interaction.

Furthermore, the MIT Taleblazer research team is exploring the prospect of adding a multiplayer option [64]. This option will take the software to another level since it will enable creators to design games involving multiple players that share the same experience by interacting, collaborating, and competing in a single game world. These multiplayer affordances will allow the re-design of the Doltso application to include new features that enhance collaboration and interaction among users.

## 9. Conclusions

This study presents a location-based augmented reality application with gamification elements for cultural heritage communication and education. We believe this study contributes to the existing literature in the following ways:

- It presents an AR location-based application for cultural heritage communication and education and the steps followed for developing the application. In this way, the study also aims to inspire educators wishing to create their own AR tours and provide them with ideas and guidance on how to proceed with such tasks.
- It presents a method, using a VR Google Earth project, for repeating the cultural tour remotely, consolidating in this way the knowledge acquired during the AR experience.
- It proposes an evaluation scheme for location-based AR educational tours and presents descriptive statistical analysis results from this evaluation.
- It presents the results of a structural equation modeling analysis, determining the causal relationships between the evaluation factors.

More specifically, the study presents the stages for implementing the application, emphasizing certain decisions made in the requirements analysis and application design phase. The application’s target audience is mainly primary and secondary education students following a specific educational program.

The application guides the users through a city district with historical and cultural interest where many old constructions, mainly from the Byzantine and post-Byzantine eras, are preserved. During the AR tour, the users are prompted to take on different roles (e.g., tourist, architect, resident of the area, and active citizen), observe their surroundings, and answer questions related to these roles. A Google Earth interactive web application was also developed in order to be utilized after the AR tour for revising and consolidating the acquired knowledge.

In order to evaluate the application, an evaluation scheme was developed mainly by using constructs derived from the related literature. A sample of 309 students evaluated the AR application. The findings show that the students felt intense feelings of challenge and perceived the application as highly useful in terms of knowledge gains. Furthermore, structural equation modeling analysis revealed the causal relations among the study’s constructs. The results from the structural equation analysis showed that the constructs of challenge and users’ interaction/collaboration had a significant and positive impact on the users’ perceived knowledge. It can also be argued that although challenge and interaction did not have direct effects on re-usage intentions, they had in-direct effects through the construct of educational usefulness/knowledge, which can be regarded as an important mediator of the above relationships.

## Figures and Tables

**Table 1:** Mean scores, standard deviation, and standardized coefficients.

	Mean Scores	Standard Deviation	Standardized Coefficients
**Challenge**			
I feel proud when I advance with success in the game “Doltso”	4.17	0.93	0.82
It feels rewarding to get to the next level of the game	4.17	0.93	0.86
I feel excited when I answer a question correctly	4.29	0.95	0.69
**Usefulness/Knowledge**			
The application improves my knowledge about the cultural heritage of my city	4.16	0.91	0.73
The application helps me to get to know better the district of Doltso	4.22	0.93	0.64
The application motivates me to obtain new knowledge	3.98	0.99	0.85
**Interaction/Collaboration**			
The game Doltso is an opportunity for collaboration	4.00	0.96	0.85
I was collaborating with my friends while using the application	3.68	1.20	0.85
**Intention to reuse**			
I intent to play the game again in the near future	3.37	1.28	0.84
I would like to play a similar game again in the future for another district of the city.	4.12	1.03	0.77

**Table 2:** Discriminant validity of the study’s constructs, Notes: a: average variance extracted; b: square of correlations between factors.

	Challenge	Knowledge	Interaction	Intention
Challenge	0.627 a	0.490	0.416	0.319
Usefulness/Knowledge	0.490 b	0.558	0.448	0.543
Interaction/Collaboration	0.416	0.448	0.532	0.316
Intention	0.319	0.543	0.316	0.650

**Table 3:** Results of Hypotheses Testing (Direct Effects).

Hypothesized Relationships	Standardized Direct Coefficient	Critical Ratios (Significance)
H1: Challenge → Usefulness/knowledge	0.459	5.081 (0.000)
H2: Challenge → Interaction/Collaboration	0.645	9.566 (0.000)
H3: Challenge → Intention to reuse	0.060	0.626 (0.532)
H4: Interaction/Collaboration → Educational Usefulness/knowledge	0.374	3.719 (0.000)
H5: Interaction/Collaboration → Intention to reuse	0.105	1.069 (0.285)
H6: Usefulness/knowledge→ Intention reuse	0.624	5.585 (0.000)

**Table 4:** Indirect Effects.

	Indirect Effects (Significance)
Challenge → Usefulness/knowledge	0.209 (0.004)
Challenge → Intention to reuse	0.574 (0.003)
Interaction/Collaboration → Intention	0.247 (0.004)

**Figure A1:**
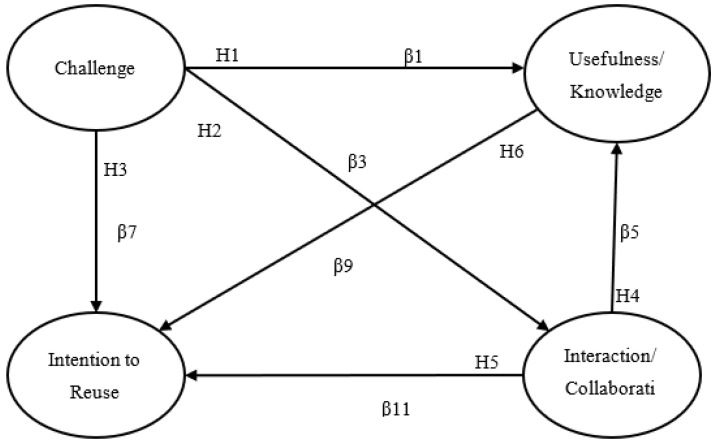
Structural Equation Modeling Analysis.

## Data Availability

Data available on request due to privacy restrictions.

## Appendix A

**Questions used in the Likert scale questionnaires (in Greek).**

**Challenge**

1. I feel proud when I advance with success in the game “Doltso”
2. It feels rewarding to get to the next level of the game.
3. I feel excited when I answer a question correctly.

**Outdoor Activity**

4. The Doltso game motivates me to explore new places.
5. The Doltso game motivates me to meet my friends and collaborate with them in the district of Doltso
6. The Doltso game is an opportunity to get out of the house and explore a traditional district of my Kastoria

**Ease of use**

7. Getting familiar with the game interface doesn’t require much effort.
8. The interaction with the game Doltso is clear and understandable
9. The Doltso game is easy to use.
10. I think that I would need the support of a technical person to be able to play the game.

**Usefulness/Knowledge**

11. The application improves my knowledge about the cultural heritage of my city
12. The application helps me to get to know better the district of Doltso
13. The application motivates me to obtain new knowledge.

**Interaction/Collaboration**

14. The game Doltso is an opportunity for collaboration.
15. I was collaborating with my friends while using the application.

**Intention to reuse**

16. I intent to play the game again in the near future.
17. I would like to play a similar game again in the future for another district of the city.

## Appendix B

**Structural Model Equations**

Usefulness = β0 + β1 (Challenge) + ε1

Interaction = β2 + β3 (Challenge) +ε2

Usefulness = β4 + β1 (Challenge) + β5 (Interaction) + ε3

Intention: β6 + β7 (Challenge) + ε4

Intention: β8 + β9 (Usefulness/knowledge) + ε5

Intention: β10 + β11 (Interaction/Collaboration) + ε6

Usefulness = β12 + β5 (Interaction/Collaboration) + ε7

Intention = β13 + β7 (Challenge) + β9 (Usefulness/Knowledge) + ε8

Intention = β14 + β11 (Interaction/Collaboration) + β9 (Usefulness/Knowledge)
